# “Push it!” or “Hold it!”? A comparison of nicotine-avoidance training and nicotine-inhibition training in smokers motivated to quit

**DOI:** 10.1007/s00213-021-06058-5

**Published:** 2022-01-11

**Authors:** Alla Machulska, Mike Rinck, Tim Klucken, Kristian Kleinke, Jana-Carina Wunder, Olga Remeniuk, Jürgen Margraf

**Affiliations:** 1 Department of Clinical Psychology, Institute of Psychology, University of Siegen, Adolf-Reichwein-Str. 2a, D–57068 Siegen, Germany; 2 Behavioural Science Institute, Radboud University Nijmegen, Nijmegen, The Netherlands; 3 Mental Health Research and Treatment Center, Ruhr-Universität Bochum, Bochum, Germany

**Keywords:** Cigarette smoking, Smoking cessation, Nicotine addiction, Approach bias, Approach bias modification, Inhibition training, Approach-Avoidance-Task, Inhibitory training

## Abstract

**Rationale:**

Recently, experimental paradigms have been developed to strengthen automatic avoidance or inhibitory responses for smoking cues. However, these procedures have not yet been directly compared regarding their effectiveness and mechanisms of action.

**Objective:**

This study compared the effects of avoidance vs. inhibitory training as an add-on to a brief smoking cessation intervention. The standard Approach-Avoidance-Task (AAT) was adapted for both training types and control conditions.

**Methods:**

One hundred twenty-four smokers attended behavioral counseling for smoking cessation and were thereafter randomized to one of four training conditions: avoidance-AAT, sham-avoidance-AAT, inhibition-AAT, sham-inhibition-AAT. During a 2-week training period including five training sessions, smokers in the avoidance-AAT trained to implicitly avoid all smoking-related cues, while smokers in the inhibition-AAT trained to implicitly inhibit behavioral response to smoking cues. During sham training, no such contingencies appeared. Self-report and behavioral data were assessed before and after training. Cigarette smoking and nicotine dependence were also assessed at 4- and 12-week follow-ups.

**Results:**

At posttest, avoidance training was more effective in reducing daily smoking than inhibition training. However, this difference was no longer evident in follow-up assessments. All training conditions improved other smoking- and health-related outcomes. Neither training changed smoking-related approach biases or associations, but approach biases for smoking-unrelated pictures increased and Stroop interference decreased in all conditions. Smoking devaluation was also comparable in all groups.

**Conclusions:**

Avoidance training might be slightly more effective in reducing smoking than inhibitory training. Overall, however, all four training types yielded equivalent therapy and training effects. Hence, a clear preference for one type of training remains premature.

**Supplementary Information:**

The online version contains supplementary material available at 10.1007/s00213-021-06058-5.

Although most smokers are fully aware of the devastating effects associated with cigarette smoking, nicotine dependence continues to be one of the most frequent substance use disorders worldwide. Concurrently, possibly due to the current COVID-19 pandemic, awareness of smoking risks and the willingness to stop smoking in some groups of smokers have increased recently (Kayhan Tetik et al. 2021; Kowitt et al. 2020), rendering it important to optimize existing interventions for smoking cessation.

Proven interventions for smoking cessation include educational approaches, behavioral therapies and counseling, nicotine replacement therapies, and other approved medications (Batra and Petersen 2015; Hajek et al. 2013; Hopkins et al. 2001). However, relapse rates are still high and sustained abstinence is difficult to achieve (Cummings and Hyland 2005; Holmes et al. 2004). This might be explained by the fact that common interventions often fail to tap into more automatic, hard-to-control processes, which have been implicated in substance use. Dual-process models of addiction (Wiers et al. 2007) constitute a valuable theoretical framework for this notion and assume that both reflective and impulsive processes drive individual choices and behavior. Reflective processes comprise executive cognitive functions such as response inhibition and interference control (Gray and McNaughton 2000), while impulsive processes include implicit associations and automatic approach-avoidance tendencies. In the course of addiction, the balance between impulsive and reflective processes appears to be severely disturbed. Specifically, repeated drug use has been shown to strengthen impulsive processes, thereby leading to the preferred processing of drug cues over other cues in the environment (MacLeod and Mathews 2012). At the same time, reflective processes are weakened through acute and chronic drug effects, impeding resistance to use addictive substances (Hofmann et al. 2009).

The automatic tendency to approach drug-related cues can be viewed as a manifestation of strengthened impulsive processes and has been repeatedly implicated in unhealthy consumption behavior (i.e., Kemps and Tiggemann 2015), including cigarette smoking (Machulska et al. 2015; Watson et al. 2013; Wiers et al. 2013). An encouraging finding in recent years is that maladaptive approach biases for drug cues appear to change as a result of repeated training (for a review, see Kakoschke et al. 2017). This in turn has been linked to reduced consumption behavior (Machulska et al. 2016) and less relapse (Wiers et al. 2011; Eberl et al. 2013). The Approach-Avoidance-Task (AAT; Rinck and Becker 2007) appears to be a feasible way to both measure and modify approach biases for drug-related cues. During this task, participants react to pictures presented on a computer screen by making pull (approach) or push (avoidance) movements. While the assessment version of the task involves an equal number of pulling and pushing trials, the training version requires to push most or all drug-related images and to pull most or all alternative images. Thus, the latter constitutes drug-avoidance training.

In contrast, the ability to withhold a behavioral response is a key aspect of reflective processes as it provides time for a more deliberate decision based on long-term consequences, norms, and values (Logan et al. 1984). Deficits in inhibitory control can lead to loss of control of substance use, which then can lead to the manifestation of substance use disorders (APA 2013). In the context of cigarette smoking, the quality of inhibition control predicted the ability to initiate or maintain abstinence following a quit attempt (Krishnan-Sarin et al. 2007). A meta-analysis comprising 97 studies revealed that inhibitory deficits are most strongly apparent for psychostimulants, including smoking (Smith et al. 2014). As with approach biases, inhibition skills seem to be malleable through training (Klingberg 2010). Inhibitory control can be measured and trained with a variety of computerized tasks, with the Go/No-Go (GNG) task (Newman and Kosson 1986) being one of the most frequently used tasks in this context. In the GNG task, go-cues (i.e., a circle) or no-go-cues (i.e., a triangle) are presented on a series of pictures, and participants are instructed to press a button whenever a go-cue is present and to withhold that response whenever a no-go-stimulus is displayed. During inhibitory control training using the GNG task, no-go-cues are constantly paired with substance-related pictures. Applying the GNG-paradigm to hazardous drinkers, Houben and colleagues (Houben et al. 2012) showed that pairing alcohol cues with no-go signals led to reductions in alcohol intake. A similar finding was reported for the eating domain (Lawrence et al. 2015). Additionally, two meta-analyses confirmed that inhibition training with the GNG task led to robust reductions in food and alcohol consumption (Allom et al. 2016; Jones et al. 2016). Although applications to cigarette smoking are scarce, a recent training program provided evidence that training smokers to repeatedly inhibit a response toward smoking cues led to benefits in maintaining abstinence as compared to sham training (Adams et al. 2017).

Taken together, the last decade was characterized by numerous efforts to contribute positively to impulsive and reflective processes implicated in addiction, either by reducing maladaptive approach biases or by strengthening inhibition control. More specifically, both types of training are aimed at altering participants’ automatic (habitual) response in order to foster more adaptive responses when they are encountered with smoking cues. If effective, both trainings should transfer to participants’ daily operational routine, meaning that when smokers are exposed to cigarettes or associated cues, the training should enable them to either reject (avoidance) or ignore (inhibition) such cues. However, the exact working mechanisms remain poorly understood. While some evidence hints to the fact that reductions in approach bias mediate training outcomes (Eberl et al. 2013; Wiers et al. 2011), some authors fail to find such links, and other report sham training to be equally effective (Preis et al. 2020; Wittekind et al. 2019). In the context of inhibition training, Veling et al. (2008) showed that clinical improvements were mediated by a devaluation of no-go stimuli. Although some findings support this notion (Houben et al. 2012), some conflicting results have also been reported (Jones et al. 2016). Finally, although previous evidence suggests that both training versions (avoidance or inhibition) seem to be effective in clinical settings, it is unclear whether one type of training is preferable over the other. Hence, the effects contributed to approach bias retraining or inhibition training have not yet been compared to each other in the realm of nicotine addiction (but see Di Lemma and Field, 2017, for a comparison of both types of training in the context of alcohol consumption).

The present study aimed at translating the rationale for a head-to-head comparison of approach bias retraining and inhibition training to the context of tobacco smoking. Our objective was to address former shortcomings in the literature. To this end, smokers motivated to quit smoking were subjected to multiple (avoidance or inhibition) trainings that were embedded into a regular smoking cessation intervention. Various experimental paradigms and measurements were employed to test for direct training effects as well as for close and far generalization. Using adequate control groups (sham avoidance and sham inhibition training), we expected the active conditions to be more effective in reducing cigarette smoking and in contributing to related changes in smoking behavior. Our secondary aim was to investigate mechanisms of action by examining whether the active trainings would result in reduced approach biases for smoking cues, improved inhibition control, and/or devaluation of smoking cues. The standard joystick-based AAT was adapted for both approach bias assessments, as well as approach bias modification and inhibition training.

## Methods

### Participants

We recruited 124 smokers (69 females, 55 males) from the general population and from students at Ruhr-University Bochum via advertisements on websites and flyers. Interested participants took part in a telephone interview and were screened for eligibility criteria. Participants were included if they were aged 18 years or older, smoked at least six cigarettes per day during the last 6 months, and reported a motivation to quit smoking. Exclusion criteria were current psychiatric illness, including other substance use disorders, insufficient German language skills, uncorrected visual or auditory impairment, and dyschromatopsia. Full written informed consent was obtained from each participant at study entry. Participation was voluntary and participants had the right to withdraw their consent at any time. Subjects were not paid; however, student participants received course credit for participation.

### Ethics statement

The study protocol was approved by the local Ethics Committee of the Ruhr-University of Bochum and was conducted in accordance with the Declaration of Helsinki and Good Clinical Practice guidelines.

### Trial design

A mixed design was employed. The between-subjects variable was training condition, as participants were randomly assigned to one of four groups: (1) Avoidance-AAT (A-AAT), (2) sham Avoidance-AAT (sham A-AAT), (3) GNG-AAT, or sham (4) GNG-AAT. Time constituted the within-subjects variable. An extensive set of behavioral (reaction time) and self-report data were assessed before (pretest) and after (posttest) training.

The statistical power achieved with the final sample size (114 divided into 4 groups, see below) was computed using the program G*Power 3.1 (Faul et al. 2009). The power to detect medium-sized group-by-time 4 × 2 interactions (*f* = 0.25, with *p* = 0.05 and *r* = 0.50) was excellent (*1-ß* > 0.99). It was adequate (*1-ß* = 0.81) for small-to-medium interaction effects of *f* = 0.16 and insufficient (*1-ß* = 0.39) to detect small interaction effects of *f* = 0.10.

In addition, training effects (approach-avoidance or GNG-biases) and cigarette craving were measured prior to each training session, while a number of cigarettes smoked daily and degree of nicotine dependence were also assessed at 4- (FU-4) and 12-week follow-ups (FU-12). Participants were blind to experimental conditions (active or sham training). Due to the study design, experimenters could not be blinded.

### Primary and secondary outcome measures

Our primary outcome measure comprised reductions in daily cigarette consumption. Secondary outcome measures were reductions in other smoking-related variables (i.e., craving, motivation to quit, nicotine dependence, devaluation of smoking) and changes in reaction time-based measures (motor approach-avoidance biases, approach-avoidance associations, GNG-biases, Stroop effects). Exploratory analyses were conducted in order to investigate whether changes in biases were associated with reduced smoking. To account for possible adverse effects or symptoms shifting, alcohol consumption and psychological functioning (depression, anxiety, and stress) were also assessed. Primary timepoints for analyses were changes from pre- to post-assessment. Secondary timepoints included between-session measures and FU assessments.

### Procedure

**Fig. 1:**
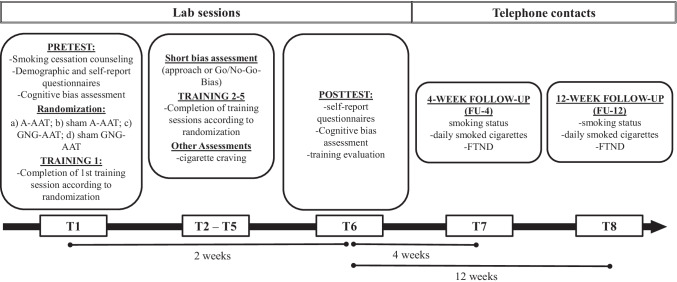
Experimental procedure

In session 1, participants provided informed consent, took part in a brief smoking cessation counseling, completed a carbon monoxide (CO) breath test, cognitive bias assessments, and questionnaire measures (pretest). Thereafter, smokers were randomized to one of the four training conditions. The first training session was carried out at the end of the first session. Trainings were then repeatedly administered at sessions 2–5, resulting in five training sessions. Prior to each training session, participants were required to indicate their craving levels and they completed a short bias assessment (approach bias assessment or GNG-bias assessment, depending on training variant). After completion of the final training session, participants again completed the CO breath test, answered questionnaires, and performed cognitive bias assessment tasks (posttest). In addition, participants evaluated the trainings and indicated their awareness about training contingencies. Hence, six laboratory sessions were accomplished during a 2-week interval. Finally, participants were contacted by telephone at 4 (FU-4) and 12 weeks (FU-12) after the final laboratory session. They were asked to indicate their smoking status, the number of cigarettes smoked daily, and their degree of nicotine dependence. Figure 1 displays a schematic overview of the experimental procedure.

### Interventions

#### Smoking cessation counseling

Prior to randomization, all smokers took part in a brief behavioral counseling for smoking cessation (approximately 90 minutes). Afterwards, smokers received a self-help book (a German copy of “The Easy Way to Stop Smoking” by Allen Carr) to aid smoking cessation, and they were instructed to self-monitor daily smoking by means of a smoking diary.

#### AAT trainings

##### General training design

During training, different nicotine-related and shape- and color-matched tooth-cleaning (control) pictures were successively presented on a computer screen. Both picture categories were derived from Stippekohl and colleagues (Stippekohl et al. 2010). Nicotine-related pictures comprised close-up photographs of individuals smoking, lighting, or taking a cigarette out of a package. Tooth-cleaning pictures displayed the same individuals brushing their teeth or taking a toothbrush out of a toothbrush holder. Each picture category contained 10 different images. A joystick (Logitech Extreme 3D) was attached to the computer and participants were required to respond as quickly and accurately as possible to picture orientation by joystick movements. Upon a false reaction, an arrow-feedback appeared on the computer screen and participants had to correct their movement to make the picture disappear. Each training session started with 40 test trials (bias assessment) and continued with 200 training trials. Participants were allowed to take a short break halfway through. Training sessions took approximately 15 minutes to complete.

##### A-AAT

Pictures were tilted either 3° to the left or 3° to the right. Participants were instructed to ignore picture content and to respond to picture orientation by pulling closer all images rotated to the left and pushing away all images rotated to the right using the joystick. As a result, pulled images grew in size, while pushed images shrank. While nicotine-related and control pictures had to be pulled and pushed equally often during the 40 test trials, unbeknown to the participants, a contingency between picture content and arm movement was introduced for training purposes: During training, all nicotine-related pictures were rotated 3° to the right and had to be pushed, meaning that this variant constituted a nicotine-avoidance training. Accordingly, all tooth-cleaning pictures were rotated 3° to the left and had to be pulled. Each nicotine-related picture was presented 10 times in push-away format, while each tooth-cleaning picture was presented 10 times in pull-closer format, resulting in 200 training trials.

##### Sham A-AAT

The training was similar to the A-AAT with the exception that no contingency between image content and arm movement (pull vs. push) was introduced. Each nicotine-related and tooth-cleaning picture was presented five times in push-away format and five times in pull-closer format, resulting in 200 training trials. Hence, this training variant resembled a prolonged approach bias assessment test.

##### GNG-AAT

For inhibition training, pictures were again tilted 3° to the left or right. Unlike the avoidance training, untilted images were also presented. Participants were instructed to react to rotated images (independent of tilt direction) by moving the joystick to the left and to withhold their reaction to untilted images until a 2000 millisecond (ms) timeout had elapsed and the picture disappeared on its own. The training started with 40 test trials, where nicotine-related and control pictures were equally often presented in go (tilted) or no-go (untilted) format. Unbeknown to the participants, a contingency was introduced during the training trials, meaning that all nicotine-related pictures were not tilted (no-go format), and therefore, participants had to withhold their reaction. Accordingly, all tooth-cleaning images were tilted to the left or to the right (go format) and had to be reacted to. Each nicotine-related picture was presented 10 times in no-go format, while each tooth-cleaning picture was presented 10 times in go format, resulting in 200 training trials in total. As such, this training variant constitutes nicotine-inhibition training. The AAT-based inhibition training lacked the typical approach-avoidance movement (arm flexion vs. extension) and zooming-effect but otherwise applied comparable task requirements in terms of instructions (i.e., ignore picture format, react to picture orientation) and response modality (joystick movement). Doing so, we maximized the similarity of non-specific training variables and only allowed experimental variation concerning the crucial training mechanisms (avoidance vs. inhibition). Consequently, differences in training or therapy effects should be attributed to differences in the process at target, as the influence of confounding factors was controlled for.

##### Sham GNG-AAT

The training was similar to the GNG-AAT with the exception that no contingency between image content and reaction (react vs. inhibit) was introduced. Each nicotine-related and tooth-cleaning picture was presented five times in no-go and five times in go format, resulting in 200 training trials. Hence, this training variant resembles a prolonged GNG-bias assessment test.

### Assessments

#### Cognitive bias assessment

Each task was presented on a personal computer using Inquisit 4 Lab software, except for the AAT, which was programmed in Microsoft Visual Basic. Estimates of reliability (internal consistencies) were calculated for each task and measurement time point (see Supporting information Appendix, Table S1). Please advise detailed supplementary materials for detailed task descriptions.

##### Motor approach biases for smoking (training effects and close generalization)

Prior to the first (pretest) and upon the last (posttest) training session, all smokers completed an assessment version of the AAT (based on Machulska et al. 2016). For the purpose of bias assessment, 15 nicotine-related and 15 tooth-cleaning (control) pictures had to be pulled and pushed equally often. Two-thirds of the pictures of the assessment AAT were also used in the training-AATs. The remaining pictures were not presented during training, which was done to allow for a test of close generalization effects. Images were rotated either 3° to the left or 3° to the right. Similar to the training, participants were instructed to pull images rotated to the left and to push images rotated to the right. Each image was presented three times in pull-closer and three times in push-away format, resulting in 180 test trials. In addition, participants randomized to the A-AAT (active or sham condition) completed a brief approach bias assessment consisting of 40 test trials prior to each training session. An approach bias score was calculated for each participant and picture category by subtracting the median reaction time (RT) for pulling the picture category from the median RT for pushing the picture category (see Rinck and Becker 2007). By doing so, a positive score indicates an approach tendency toward a picture category, whereas a negative score indicates an avoidance tendency.

##### Approach associations for smoking (broad generalization)

All participants completed a single-target Implicit-Association-Test (st-IAT) at pre- and posttest. The st-IAT was based on Woud and colleagues (Woud et al. 2016). The task started with an attribute discrimination block, in which participants had to categorize six approach- or avoidance-related words by pressing a keyboard key. The first combined block (24 practice + 72 test block trials) added six different smoking words. During the compatible block assignment, smoking and approach-related words shared a response key, while in the incompatible block (24 practice + 72 test block trials), smoking and avoidance-related words shared the same response key. During each trial, reminder labels remained visible on the computer screen. Upon incorrect responses, a red “X” appeared in the center of the screen. Response assignments and block order were counterbalanced across participants. An approach bias was calculated by subtracting the median RT of the compatible block from the median RT of the incompatible block. Accordingly, a positive score reflects stronger approach associations toward smoking words.

##### Nicotine-related response inhibition (close generalization)

Each GNG training session started with 40 test trials, in which smoking and control images were equally often presented in go- and no-go format. Hence, participants had to respond or withhold their response to nicotine-related and control pictures with equal frequency. According to Di Lemma and Field (2017), a GNG-bias can be inferred from subtracting the RTs for go-smoking trials from those of go-control trials. A positive score indicates that smokers are faster to respond to smoking pictures than to control pictures, suggesting an automatic tendency to react to smoking cues.

##### Inhibition of habitual responses (broad generalization)

All participants performed the classical Stroop task (Stroop 1935), which was adapted from Hepp and colleagues (Hepp et al. 1996). Stimuli consisted of color words (German equivalents for “red,” “green,” “blue,” and “black”) and neutral objects (rectangles), all of which were printed in either red, green, blue, or black. Participants were required to ignore the semantic meaning of the words and instead indicate the print color by responding via keyboard button presses. During each trial, reminder labels remained visible on the computer screen. Upon incorrect responses, a red “X” appeared in the center of the screen. During congruent trials (28 trials), color words appeared in their corresponding color. In incongruent trials (28 trials), color words were printed in a non-matching color. Neutral control trials (28 trials) consisted of colored rectangles, lacking potential matches or mismatches between semantic and visual proceedings. Following Hepp and colleagues (Hepp et al. 1996), an interference score was calculated as the difference between the median RTs of incongruent trials and neutral control trials.

#### Self-report data and biochemical verification

A broad set of questionnaires was used to access smoking behavior: Participants indicated the number of cigarettes smoked daily. In addition, exposure to nicotine was measured by means of a CO breath test (piCO™ Smokerlyzer®; Bedfont Scientific Ltd.). Craving was assessed on a 6-point Likert scale, ranging from 0 (“not at all”) to 5 (“very high). The degree of nicotine dependence was measured by means of the Fagerström test for nicotine dependence (FTND; Heatherton et al. 1991; German version: Bleich et al. 2002). Motivation to cease smoking was measured by means of the Stages of Change Scale (SoC; Prochaska et al. 1991; German version: Jäkle et al. 1999), and the Thoughts About Abstinence Scale (TAA; Hall et al. 1990). The SoC assigned smokers to different time intervals of change based on the transtheoretical model of change by Prochaska and DiClemente (1982). The TAA asked participants to select one of six abstinence goals (from 1) total abstinence, never use again, to 6) no goal at all) and required them to rate (a) their desire to quit, (b) the expected success in quitting, and (c) the expected difficulty of quitting on 10-point Likert scales. To assess smoking devaluation, participants evaluated a selected set of pictures presented during approach bias assessment according to three different criteria: valence (positive vs. negative), arousal (calming vs. arousing), and craving (highly triggering craving vs. not at all triggering craving). Again, 10-point Likert scales were applied. Two-thirds of the pictures were also presented during training, while the remaining pictures were not. This approach allows for a test of close generalization effects of AAT training on subjective stimulus evaluations. Explicit attitudes toward smoking were measured via a set of eight semantic differential items, which were based on Swanson and colleagues (Swanson et al. 2001). A 7-point Likert scale ranging between − 3 and + 3 was used to rate 8 different polar-opposite adjective pairs (i.e., healthy-unhealthy). To control for preexisting differences in substance use behavior other than smoking and in mental health, and to monitor possible adverse training effects, the Alcohol Use Disorders Identification Test (AUDIT; Saunders et al. 1993) and the Depression-Anxiety-Stress-Scale 21 (DASS 21G; Lovibond and Lovibond, 1995) were administered. All questionnaire measures were administered at pre- and posttest. The number of cigarettes smoked daily and nicotine dependence were additionally assessed at FU-4 and FU-12. Craving was measured at pretest, prior to each training session (t1, t2, t3, t4, t5) and at posttest.

#### Manipulation checks

##### Training evaluation

At posttest, participants were asked to evaluate their training and to indicate their awareness of training contingencies. The training evaluation questionnaire comprised 10 items (i.e., “In my opinion, the training exerted beneficial effects on my smoking behavior.”) with the sum score ranging between 0 and 10. This served to test whether the different trainings produced comparable expectancy effects.

##### Book reading

After training, participants were asked whether they have read the self-help book. As trainings should serve as an add-on to behavioral interventions, this manipulation check should indicate the extent to which participants made use of the behavioral advice provided by the present program.

### Data preparation and planned analysis

Missing values were replaced through multiple imputations and the intention-to-treat (ITT) principles (Fergusson et al. 2002). Prior to the computation of cognitive bias scores and Stroop scores, error trials were excluded. Participants with extremely high error rates (> 25%) were excluded from the analysis of interest. Median reaction times were used to minimize the influence of outliers (Becker et al. 2015).

Changes in cognitive biases (approach biases derived from the AAT, approach associations derived from the st-IAT) and inhibition control (GNG-biases derived from the GNG-AAT and interference scores derived from the Stroop task), as well as therapy effects, including daily smoked cigarettes, expired CO, nicotine dependence, attitudes toward smoking, and motivation to cease were analyzed by mixed ANOVA models. The Greenhouse–Geisser method was used to correct for violations of the sphericity assumption. Changes in cigarette craving were analyzed by a growth curve model (Bollen and Curran 2006). Explorative regression analyses were conducted to determine whether a change in biases was related to self-reported behavioral changes.

## Results

### Participants’ characteristics

**Fig. 2:**
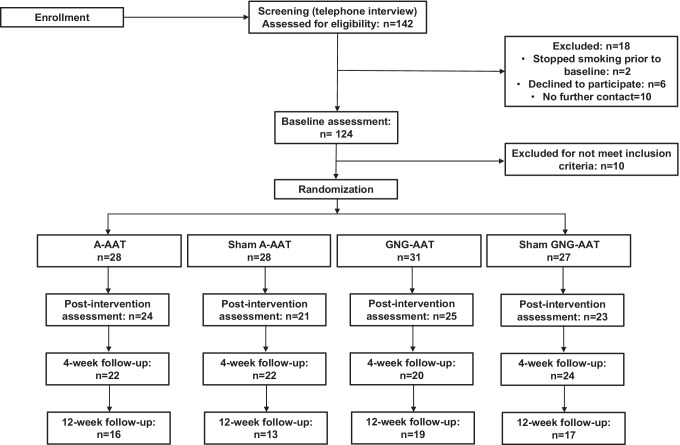
CONSORT flow diagram

A flowchart of the study participants is shown in Fig. 2. Ten participants had to be excluded for not meeting the eligibility criteria. Thus, our final sample comprised 114 participants. Baseline group differences were analyzed with univariate ANOVAs. After applying the Bonferroni-correction to account for multiple testing, no significant group differences emerged with respect to demographic, smoking-related or other psychological variables (*Fs* < 3.05, *ps*_*(uncorrected)*_ > 0.026). Table 1 provides details on baseline variables per condition.

**Table 1:** Demographic, smoking- and health-related characteristics at baseline

Variable	A-AAT (*n* = 28)	Sham A-AAT (*n* = 28)	GNG-AAT (*n* = 31)	Sham GNG-AAT (*n* = 27)	*p*
Age (years)	35.61 (11.87)	30.71 (11.42)	39.30 (15.23)	36.67 (13.88)	.103
Gender (% female)	46	36	68	63	.054
Years smoked	19.68 (11.36)	13.54 (10.28)	21.40 (14.60)	19.59 (13.00)	.095
Number of prior quit attempts	3.02 (2.73)	3.32 (2.85)	3.37 (3.16)	7.43 (18.86)	.271
CO-level	21.55 (12.57)	17.93 (9.85)	18.86 (7.34)	20.04 (12.96)	.661
Cigarettes smoked/day	18.11 (6.79)	17.04 (5.70)	19.84 (9.37)	16.04 (7.36)	.253
Cigarette craving	2.04 (1.50)	2.57 (1.29)	1.74 (1.55)	2.56 (1.25)	.068
FTND	5.04 (2.27)	4.50 (2.22)	4.45 (2.61)	4.52 (2.59)	.815
Smoking attitude	− .40 (.70)	− .49 (.83)	− 1.00 (.88)	− .78 (.88)	*.026*
Stages of Change	1.36 (.56)	1.36 (.73)	1.48 (.68)	1.41 (.64)	.861
Thoughts about abstinence					
Abstinence goal	4.07 (.54)	3.89 (.79)	4.10 (.87)	3.85 (.99)	.577
Desire to quit	8.75 (1.32)	8.61 (1.34)	8.32 (1.87)	8.33 (1.44)	.651
Anticipated success	6.14 (2.22)	6.04 (2.29)	5.97 (2.48)	6.48 (2.21)	.845
Anticipated difficulties	8.21 (1.83)	8.11 (2.06)	6.97 (2.64)	8.30 (1.61)	.051
AUDIT	5.50 (3.62)	7.36 (5.12)	4.29 (2.77)	7.07 (5.85)	*.033*
DASS-21					
Depression	4.39 (3.82)	4.71 (3.98)	3.06 (2.97)	4.26 (3.53)	.306
Anxiety	3.46 (2.97)	4.18 (3.52)	3.06 (2.83)	3.22 (3.07)	.541
Stress	6.86 (3.15)	6.50 (4.76)	5.68 (4.53)	5.26 (3.93)	.458
Self-help book (pages read)	100 (105)	92 (103)	101 (103)	82 (96)	.901
Completed training sessions	4.39 (.88)	4.25 (1.35)	4.29 (1.24)	4.56 (.85)	.739
Training evaluation	6.78 (2.00)	6.62 (1.66)	6.27 (1.89)	6.70 (2.18)	.801
Contingency awareness (%)	71	n.a	42	n.a	*.043*

Values are means, standard deviations are given in parentheses. *CO*, expired carbon monoxide in parts per million; cigarette craving (scale: 0–5); *FTND*, Fagerström test for nicotine dependence (scale: 0–10); smoking attitudes (scale: − 3– + 3); stages of change (scale: 0[precontemplation]–4[maintenance]); abstinence goal (scale: 0–5), desire to quit (scale: 1–10); anticipated success (scale: 1–10); anticipated difficulties (scale: 0–10); AUDIT = Alcohol Use Disorders Identification Test (scale: 0–40); DASS-21 = Depression-Anxiety-Stress-Scale 21 (each scale ranges from 0–21); The self-help book comprised 300 pages in total; training evaluation (scale: 0–10). Continuous variables were analyzed using univariate ANOVAs, F(3,110); All *p*-values are two-tailed. Standard deviations are given in parentheses

### Therapy effects

#### Cigarette consumption

**Fig. 3:**
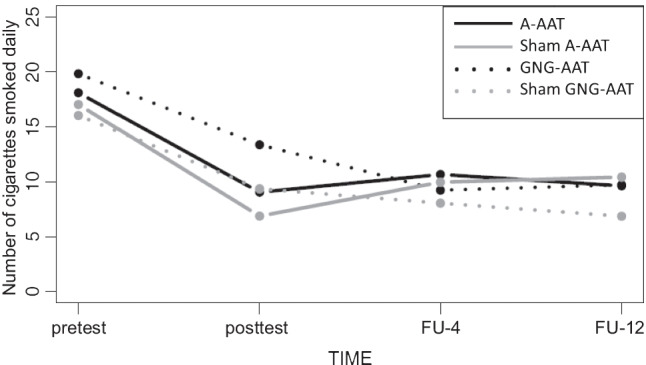
Changes in daily cigarette consumption

The 4 (training group) by 4 (time) ANOVA did not reveal a main effect for experimental condition (*F*(3, 20, 599.24) = 0.91; *p* = 0.437; see Fig. 3). There was a main effect of time (*F*(2.78, 2, 946.96) = 66.10; *p* < 0.001) and, most importantly, a condition by time interaction (*F*(8.34, 1, 620.06) = 2.28; *p* = 0.018; see Fig. 3).

The estimated marginal means (combined MI estimates) are displayed in Fig. 3. Participants in both active (A-AAT and GNG-AAT) and sham training control conditions (sham A-AAT and sham GNG-AAT) on average smoked fewer cigarettes at posttest, as well as on both FU assessments in comparison to the pre-measurement. Follow-up comparisons revealed that smokers assigned to the A-AAT group experienced a stronger reduction in daily nicotine consumption at posttest than smokers in the GNG-AAT. However, this difference was only marginally significant (*F*(1, 6, 786.05) = 3.51; *p* = 0.061). At follow-up examinations, group differences could no longer be discerned. No other between-group differences emerged.

#### Expired CO

The 4 (training group) by 2 (time) ANOVA did not reveal a main effect for experimental condition (*F*(3, 14, 111.41) = 1.80; *p* = 0.144). We found a main effect of time (*F*(1, 1, 586.18) = 27.00; *p* < 0.001) and, in line with the hypotheses, a significant condition by time interaction (*F*(3, 1, 309.31) = 2.63; *p* = 0.049). While participants in groups A-AAT (*F*(1, 918.58) = 8.28; *p* = 0.004), sham A-AAT (*F*(1, 1, 157.73) = 19.43; *p* < 0.001), and sham GNG-AAT (*F*(1, 2, 181.93) = 4.08; *p* = 0.044) experienced significantly lower CO scores at the posttest as compared to the pretest, no time-dependent reductions were found for the GNG-AAT (see Supporting information Appendix, FigA1). In addition, no significant between-group differences were found (*ps* > 0.104).

#### Cigarette craving

Cigarette craving was assessed at pre- and posttest, as well as prior to each training session. We fitted a quadratic growth curve model to the data (see Supporting information Appendix, Table S2 and FigA2). Baseline levels of craving and both the linear and the quadratic time trends were regressed on the experimental condition.

Both active and sham trainings had beneficial effects on cigarette craving. Predicted levels of craving were on average lower at the end of the follow-up phase in comparison to the pre-measurement. The small, but significant positive quadratic slope indicates that at the end of the training phase, cigarette craving started to slightly rise again.

#### Nicotine dependence

The 4 (training group) by 4 (time) ANOVA did not reveal a main effect for experimental condition (*F*(3, 22, 597.44) = 0.74; *p* = 0.529) nor a condition by time interaction (*F*(8.29, 912.91) = 0.62; *p* = 0.772). However, we found a main effect of time (*F*(2.76, 1, 367.43) = 42.20; *p* < 0.001; see Supporting information Appendix, FigA3). Participants in all conditions on average had a lower FTND score at posttest, as well as on both FUs in comparison to the pretest.

#### Motivation to cease smoking

We found main effects of time for motivation to change as assessed by the SoS (*F*(1, 2, 720.28) = 10.44; *p* = 0.001) and the TAA-subscale “expected difficulty of quitting”; *F*(1, 6, 572.02) = 12.62; *p* < 0.001; see Supporting information Appendix, Table S5). Results indicated that smokers’ readiness to change increased and anticipated difficulties of quitting decreased across time. No interaction effects were found.

#### Stimulus devaluation

The 2 (generalization: trained vs. untrained pictures) × 2 (picture category: smoking-related vs. control pictures) × time (pre vs. post) × 4 (training group) × 3 (rating: valence, craving, arousal) ANOVA yielded the following main effects and interactions: condition (*F*(3, 18, 269.25) = 3.25; *p* = 0.021), time (*F*(1, 2, 779.18) = 19.94; *p* < 0.001), rating (*F*(1.97, 2, 651.64) = 96.09; *p* < 0.001), picture category (*F*(1, 8, 613.55) = 5.46; *p* = 0.020), condition by picture category interaction (*F*(3, 9, 121.46) = 4.19; *p* = 0.006), time by picture category interaction (*F*(1, 3, 274.16) = 85.33; *p* < 0.001), time by rating interaction (*F*(1.94, 5, 003.23) = 14.22; *p* < 0.001), picture category by rating interaction (*F*(1.80, 5, 631.63) = 137.53; *p* < 0.001), time by picture category by rating interaction (*F*(1.93, 5, 556.73) = 12.28; *p* < 0.001), and condition by picture category by rating interaction (*F*(5.41, 14, 676.62) = 2.33; *p* = 0.035). Interactions are displayed in FigA7. For smoking-related pictures, there was a small decrease in arousal ratings from pre to post in both A-AAT conditions and the sham GNG-AAT condition, while in the GNG-AAT, a slight increase in arousal ratings appeared. Furthermore, there was a considerably larger decrease in valence and craving ratings independent of the experimental condition. With regard to tooth-cleaning pictures, there was a slight increase in arousal ratings from pre to post in all conditions. Valence ratings also slightly increased from pre to post in all conditions but the GNG-AAT, while for the GNG-AAT, there is a slight decrease of about the same size. Regarding craving, there is virtually no change for the active conditions (A-AAT and GNG-AAT), while for both sham conditions, craving slightly increases from pre to post. Finally, we want to comment on some general patterns: smoking-related pictures generally received lower valence ratings than the control pictures. This effect was more pronounced at the post-measurement time point. Furthermore, initially, smoking-related pictures received higher craving ratings in comparison to the control pictures. This effect diminished throughout the intervention phase. Finally, given that no main effects or interactions appeared for the factor “generalization,” all changes reported above are not restricted to pictures that were only presented during training, suggesting a close generalization effect of AAT training on stimulus evaluation.

#### Smoking attitudes

For attitudes toward smoking as assessed by means of the semantic differential, we found a main effect of group (*F*(3, 12, 588.90) = 3.53; *p* = 0.014), suggesting that participants in the avoidance training groups (A-AAT and sham A-AAT) had slightly less positive attitudes toward smoking than smokers in the inhibition groups (GNG-AAT and sham GNG-AAT). Furthermore, we found main effects of time (*F*(1, 2, 099.43) = 45.74; *p* < 0.001; see Supporting information Appendix, Table S4), indicating that in general, attitudes toward smoking became more negative. No interaction between condition and time emerged.

#### Alcohol consumption and psychological functioning

For alcohol use, there was a main effect of group, indicating that alcohol use was more pronounced in the sham groups (sham A-AAT and sham GNG-AAT) as compared to the experimental groups (*F*(3, 207, 390.92) = 2.70; *p* = 0.044; see Supporting information Appendix, Table S5). Baseline levels regarding general psychopathology were comparable across groups. We found main effects of time for alcohol use (*F*(1, 1, 269.38) = 11.51; *p* = 0.001), as well as general psychopathology in terms of stress (*F*(1, 9, 946.34) = 7.76; *p* = 0.005), anxiety (*F*(1, 5, 046.75) = 17; *p* < 0.001), and depression (*F*(1, 5, 415.91) = 15.91; *p* < 0.001; see Table S5). Alcohol use, levels of stress, anxiety, and depression decreased over time. No interaction effects were found.

### Training effects

#### Changes in approach biases

##### AAT

**Fig. 4:**
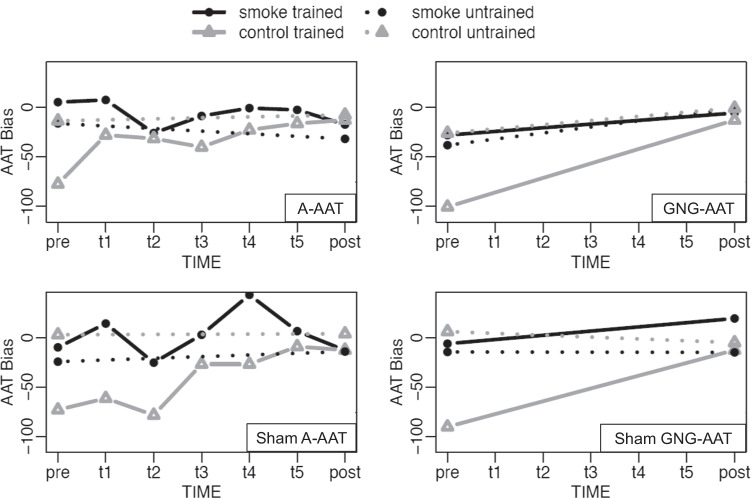
Changes in approach biases

The 4 (training group) by 2 (time: pre vs. post) by 2 (generalization: trained vs. untrained pictures) by 2 (picture category: smoking-related vs. control pictures) ANOVA did not reveal a main effect for experimental condition (*F*(3, 68, 835.74) = 0.12; *p* = 0.949). There were main effects for time (*F*(1, 6, 732.61) = 7.10; *p* = 0.008) and for generalization (*F*(1, 5, 072.62) = 14.20; *p* < 0.001), indicating that approach bias scores were larger for post- than pretest and for stimuli which have been used for assessment purposes only. In addition, there was a main effect for picture category (*F*(1, 1) = 12.16; *p* < 0.001), implying larger approach tendencies for smoking pictures than for tooth-cleaning control pictures and thereby replicating a nicotine-related approach bias. Furthermore, we found two-way interactions between generalization and time (*F*(1, 6, 263.59) = 4.63; *p* = 0.031), between picture category and time (*F*(1, 5, 048.82) = 5.21; *p* = 0.022), and between generalization and picture category (*F*(1, 10, 924.26) = 21.78; *p* < 0.001). Finally, there was also a three-way interaction between time, generalization, and picture category (*F*(1, 4, 578.25) = 4.70; *p* = 0.030). The interactions are illustrated in Fig. 4. For smoking-related pictures, approach biases were slightly larger for pictures presented during training than for those never shown in training sessions (assessment only). However, as indicated in Fig. 4, the slopes were quite parallel, implying that bias scores changed in approx. the same way from pre- to posttest. For tooth-cleaning control pictures, bias scores for pictures shown during training increased, while bias scores for pictures used for assessment only remained unchanged. Hence, bias change with regard to tooth-cleaning control pictures was restricted to the picture set presented during training.

##### AAT: additional analyses

As each training session was preceded by 40 test trials, approach biases for A-AAT and sham A-AAT were also available for these additional five time points. The 2 (picture category: smoking-related vs. control pictures) × 2 (training group: A-AAT vs. sham A-AAT) × 5 (time: training sessions 1 − 5) ANOVA did not reveal a main effect for experimental condition (*F*(1, 13, 753.74) = 0.17; *p* = 0.680), a main effect of time (*F*(3.13, 1, 224.95) = 1.79; *p* = 0.145) nor a condition by time interaction (*F*(3.13, 1, 605.76) = 0.77; *p* = 0.516). We found a main effect for picture category (*F*(1, 771.18) = 8.90; *p* = 0.003), again replicating a smoking-related approach bias. Estimated marginal means are displayed in Fig. 4. No further two-way or three-way interactions were found.

##### ST-IAT

The 4 (training group) by 2 (time: pre vs. post) ANOVA did not reveal a main effect for experimental condition (*F*(3, 6, 218.19) = 0.32; *p* = 0.808), a main effect of time (*F*(1, 27, 851.87) = 0.11; *p* = 0.742) nor a condition by time interaction (*F*(3, 4, 922.17) = 1.02; *p* = 0.382).

#### Changes in inhibitory control

##### GNG-bias

As each training session was preceded by 40 test trials, GNG-biases were available for smokers in the GNG-AAT and sham GNG-AAT conditions for five time points (see FigA5). The 2 (training group: GNG-AAT vs. sham GNG-AAT) by 5 (time: training sessions 1 − 5) ANOVA did not reveal a main effect for condition (*F*(1, 5, 522.32) = 0.20; *p* = 0.657), a main effect of time (*F*(2.60, 110 1, 114.87) = 1.29; *p* = 0.277) nor a condition by time interaction (*F*(2.60, 1, 786.56) = 0.96; *p* = 0.400).

##### Stroop effects

For the Stroop interference score, the 4 (training group) by 2 (time: pre vs. post) ANOVA did not reveal a main effect for experimental condition (*F*(3, 11, 761.76) = 1.94; *p* = 0.121) nor a condition by time interaction (*F*(3, 1, 873.57) = 0.79; *p* = 0.500). There was, however, a main effect of time (*F*(1, 2, 161.99) = 3.94; *p* = 0.047; see FigA6). At posttest, participants on average showed a lower inference effect in comparison to the pre-assessment.

### Mechanisms of action

In an exploratory analysis, we regressed the number of daily smoked cigarettes at posttest on changes in motor approach biases for smoking (AAT), smoking-approach associations (st-IAT), and the Stroop inference score, while controlling for baseline levels of cigarettes smoked daily. As GNG-biases were only available for the GNG-AAT and sham GNG-AAT group, we refrained from including those biases as predictors. Changes in the respective biases and/or Stroop score were quantified as the absolute difference between the pre and the post-measurements, so that higher values indicate a stronger bias reduction respectively. Negative regression coefficients thus indicate that participants with a higher bias reduction (pre-post) tend to smoke fewer cigarettes at posttest.

**Fig. 5:**
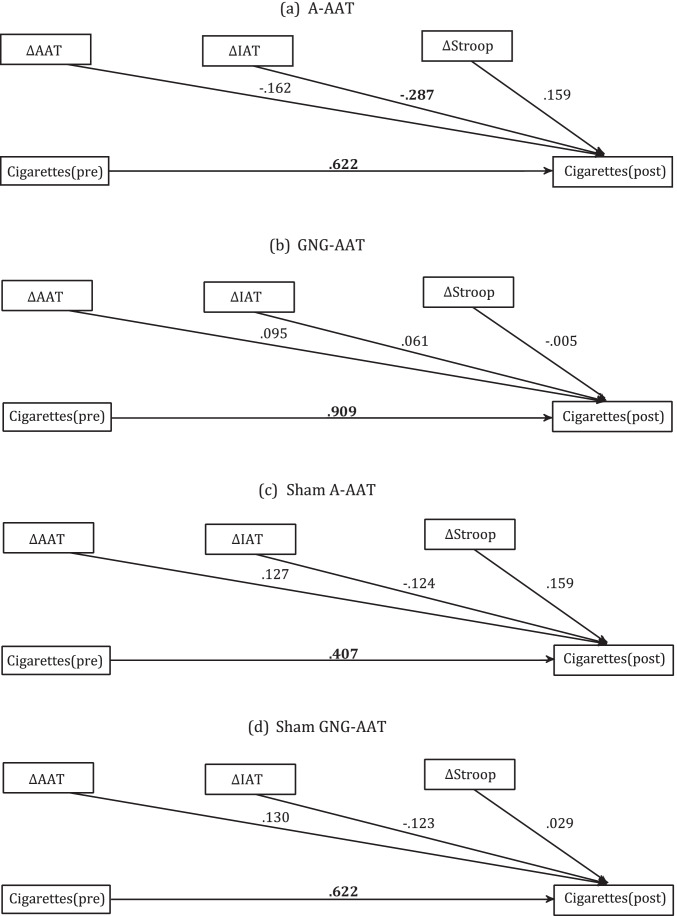
Mechanisms of action: predictors for reduced smoking

Estimated predictors are displayed in Fig. 5. In all four groups, the number of daily smoked cigarettes at the beginning of the study was the strongest predictor for the number of cigarettes smoked after the intervention phase. In contrast, changes in bias scores were not significantly related to the number of cigarettes smoked at the posttest (all *ps* > 0.05).

## Discussion

The present study aimed at investigating whether nicotine-avoidance training (A-AAT) and nicotine-inhibition training (GNG-AAT) differ in how effectively they reduce smoking behavior, as compared to accurately matched control conditions (sham training with only 50% contingency between picture content and response type). In doing so, we applied a multi-session design, which was based on different versions of the joystick training (s. Machulska et al. 2016) as an add-on to a brief behavioral counseling for smoking cessation in smokers. This randomized controlled trial (RCT) exceeds previous proof-of-principle studies and strives to amend current interventions for smoking cessation by providing additional insights into crucial factors for effective treatments. Hence, we decided to include only smokers who were motivated to quit smoking to increase the ecological validity and transferability of the results to clinical settings. To our knowledge, this is the first head-to-head comparison between avoidance and inhibition training in the realm of nicotine addiction.

Our main result revealed that at post-assessment, smokers randomized to A-AAT showed a larger reduction in the number of cigarettes smoked daily than smokers in GNG-AAT. Moreover, CO levels decreased in all groups, except in the GNG-AAT training. These findings indicate that training smokers to constantly make avoidance movements in response to smoking-related cues might be more effective than inhibitory training with regard to regular consumption behavior. However, it has to be stressed that self-reported and biochemical group differences were absent at follow-up assessments and that no other significant between-group differences appeared. Hence, the training’s advantage does not seem to be long-lasting nor definite.

The finding that group differences diminished at follow-up add to recent literature showing that the long-term effectiveness of training interventions is often limited (Machulska et al. 2021; Wittekind et al. 2019). Moreover, this calls for investigating theory-driven new approaches to improve training efficacy. One potential way to support generalization to actual smoking behavior and to ensure long-term effects is to perform trainings in multiple contexts, which ideally resemble real-world consumption conditions (Wiers et al., 2020) or to provide additional booster sessions in the longer term. Since these requirements are difficult to reconcile with laboratory training studies, a promising new approach to address these challenges could lie in providing trainings via a smartphone application (app). As most mobile phone owners carry their devices with them at practically all times, trainings could be carried out whenever convenient. In this manner, individuals can adhere to a daily training routine without time-consuming expenditure (i.e., traveling time to the lab), but also perform trainings in high-risk situations that trigger action tendencies to consume cigarettes. Indeed, more recent attempts have been undertaken to deliver psychological interventions via smartphone apps (for a review, see Zhang et al. 2018). In the realm of nicotine dependence, members of this research (Machulska et al. 2019) have developed an app-based nicotine-avoidance training and results are expected to be published in the near future. Relatedly, Wiers and colleagues (2020) refer to a new theoretical perspective based on an inferential rather than associative account and suggest special advancements for the development of effective interventions. Specifically, they conceptualize that effective training should incorporate contextual antecedents (A) that trigger adaptive behavioral (B) responses in light of goal-relevant health-related consequences (C). This means that trainings should take place in a context-relevant situation (A; i.e., high-risk smoking situation), provide a goal-relevant alternative behavior (B; i.e., approach healthy objects), and provide relevant consequences of behavioral choices (C; i.e., improved health when approaching healthy objects, deterioration of health when approaching smoking objects). Although this account awaits further investigation and empirical support from large clinical trials, it has merit in improving long-term training effects. Moreover, if applied to the current study, it becomes apparent that while our intervention fulfilled some requirements proposed by Wiers et al. (2020) such as providing a meaningful alternative behavior (B) and reflecting on relevant consequences of smoking behavior (C; see smoking cessation counseling), there is still scope for improvement.

With regard to other smoking-related outcomes (i.e., motivation to cease, cigarette craving, nicotine dependence), there was no clear superiority of training procedure. Therefore, a clear preference for avoidance training over inhibition training would be premature at this point. Although this is the first study to compare avoidance vs. inhibition training in smokers, our findings are in line with a recent study by Di Lemma and Field (2017) in the alcohol field. The authors showed that compared to sham conditions, avoidance and inhibition training both led to reduced alcohol consumption, but a clear preference of one type of training over the other could not be established.

A more straightforward outcome in the present work relates to the beneficial effect of time for almost all self-report measures, regardless of training condition. For example, cigarette consumption, craving scores, nicotine dependence, stimulus evaluation, positive attitudes toward smoking, and anticipated difficulties to cease smoking steadily decreased over time. In addition, motivation to change increased. Intriguingly, other health-related behaviors (i.e., alcohol consumption) and psychological symptoms (i.e., depression, anxiety, and stress) also decreased over time. These findings are in line with previous cognitive bias modification and executive training studies, which have also demonstrated treatment effects over time independent of whether participants received active or sham training (Adams et al. 2017; Kong et al. 2015; Machulska et al. 2021; Wittekind et al. 2019). This finding might be explained either by the fact that all participants received a brief behavioral counseling for smoking prior to randomization, which might have caused equal beneficial effects in all groups, by the design of our sham trainings, or both.

With regard to the training design, it is important to bear in mind that both sham trainings employed a 50% version of the training procedure: In the sham A-AAT condition, participants had to push half of the nicotine-related images. Likewise, participants in the sham GNG condition were required to inhibit their response to nicotine-related images in half of the trials. Moreover, smokers in all four conditions were exposed to smoking cues to the same extent, raising the question as to whether such control trainings are some sort of cue exposure paradigms. While the design of our control groups is most rigorous from an experimental perspective, as it eliminates confounds and allows for a test of how the specific training ingredients contribute to observed changes (i.e., avoidance movements, response inhibition), it might have prevented a stronger effect from being detected. As a matter of fact, the question as to whether such frequently used sham trainings truly represent control conditions or are in some way training versions themselves is the center of an ongoing debate in the literature (Blackwell et al. 2017; Lazarov et al. 2019). Obviously, the selection of appropriate comparison conditions is crucial when it comes to the interpretation of training results. Maybe the current control conditions were not sufficiently different to produce reliable differences in training effects. Hence, future studies in this context would profit from including modified or additional control conditions such as waitlist control groups or minimal substance exposure conditions (for a similar approach, see Machulska et al. 2019).

Along with beneficial treatment effects over time, we were able to observe some training effects, but again, there were no clear indications for training-specific bias changes. Most importantly, all training versions were unable to reduce smoking-related approach biases. However, a training effect appeared for tooth-cleaning control pictures: approach biases for pictures that were constantly trained to be approached increased throughout the intervention, while pictures that were only presented for assessment purposes remained unchanged. While our primary emphasis was to reduce existing approach biases for smoking by pairing smoking-related cues with constant avoidance or no-go reactions (tA-AAT), it is true that our training operates in both directions: By presenting tooth-cleaning control pictures in approach or go format, it is plausible that approach biases for those pictures increase as a result of training. However, it remains uncertain why such training effects were only found for control but not smoking pictures and why bias changes were comparable across all groups, including sham training. In line with this, the Stroop interference effect also decreased over time, with no indication for a clear training-specific effect. Finally, nicotine-related approach associations (as measured by the st-IAT) and the tendency to more rapidly react to smoking images (as measured by the assessment trials of the GNG-AAT) did not change significantly following training. Even though some studies did find reliable bias change after training, numerous other investigations were also unable to detect training-specific effects (Adams et al. 2017; Di Lemma and Field, 2017; Machulska et al. 2016; 2021). Albeit dual-process models of addiction are based on the presumption that successful bias change should precede or at least be related to behavioral changes, several reasons might account for divergent findings. Among other factors, conceptual considerations and methodological issues are among the most important. We will discuss these issues in the following.

For one part, methodological problems might explain why we did not find substantial bias reductions after training. Experimental paradigms used for bias assessment should be reliable in order to accurately measure existing biases and predict bias change over time. In fact, however, common tasks frequently suffer from methodological limitations, including poor internal consistency, temporal stability, and/or predictive validity (Ataya et al. 2012; Field and Christiansen 2012; Parsons et al. 2018). Moreover, some tasks that have been proven effective in terms of stable therapy effects (i.e., the indirect AAT: Eberl et al. 2013; Wiers et al. 2011) seem to be less suitable for bias assessment (Kersbergen et al. 2015). A close inspection of reliability indices derived from experimental paradigms used in the current study points to a large variation across tasks and measuring points (see Supplements, Table S1). While the AAT showed acceptable internal consistency (> 0.50; see Koo and Li 2017) when considering the stimulus set for training, reliability was near zero for all but one coefficient based on the stimulus set used for assessment only. The lower number of different pictures in the latter stimulus set might explain this finding. Furthermore, the GNG-bias assessment seems to be completely unreliable in this sample, rendering it relatively unlikely to detect expected bias changes over time. In contrast to that, the Stroop-related interference score as well as nicotine-approach associations derived from the st-IAT seemed to be more reliable. This, in turn, might explain why some training effects could be detected when using more reliable measures (i.e., Stroop, st-IAT). This brief analysis shows that low reliability might provide explanatory information about the failure to detect robust bias following training. At the same time, this speaks to the importance of reporting reliability estimates for each data set and experimental setup on a routine basis.

Furthermore, devaluation of smoking-related pictures as inferred from participants’ subjective picture ratings was comparable in all training groups, which might once again explain why we found time-dependent effects, but no training-specific changes in smoking-related variables. Hence, contrary to our hypotheses and unlike prior studies (i.e., Veling et al. 2008; Wessel et al. 2015), we could not observe specific effects of inhibition training on cue devaluation. The exact reasons for the failure to replicate previous findings remain elusive, but there is some evidence that devaluation appears when no-go trials are rare, but is less evident when no-go trials are frequent (Chen et al. 2016). It might be that our specific training design, which involved frequent no-go trials both in the active (100% for smoking cues) and sham (50% for smoking cues) GNG condition, prevented us from observing strong training-specific devaluation effects. Moreover, Veling and colleagues (2008) showed that devaluation was only evident for positively evaluated stimuli. It might be that for smokers who are motivated to quit and may already experience a strong ambivalence regarding smoking, such stimuli were not completely positive, to begin with, rendering it more difficult to observe training-specific devaluation (see also the incentive sensitization theory of addiction, which proposes a switch from “liking” to “wanting” as the addiction progresses; Robinson and Berridge 2008). The inspection of baseline picture ratings matches this line of reasoning, as smoking pictures received valance ratings of approximately 5 (out of 10) and were rated as less positive than tooth-cleaning control pictures.

## Limitations

In addition to notable strengths, including a multi-session design, a sample of regular smokers motivated to quit, and a well-balanced design (two active conditions with carefully matched control conditions, the use of a single apparatus, namely the AAT, for all training variants), several limitations have to be addressed. Although our sample size yielded sufficient power to detect medium-sized effects, it was insufficient for the small differences that may exist between the two active training variants investigated here. This was also true for our exploratory analyses where we found medium effect sizes (i.e., the reductions in approach associations), where the effects fell below the margin for statistical significance. Hence, replication in a larger sample would be essential to confirm or disconfirm the present results and to draw definite conclusions. Furthermore, although the decision to combine interventions based on reflective (behavioral counseling) and impulsive processes (AAT training) was guided by theoretical considerations and is most compatible with the dual-process account for addiction (Wiers et al. 2007), the present design renders it difficult to disentangle which of the observed effects are attributable to the behavioral interventions, the AAT training and/or both. To fully address this question, additional conditions (i.e., training only and counseling only) would be required.

## Conclusions

To conclude, the present study demonstrated that five sessions of avoidance or inhibition training by means of the AAT as an adjunct to behavioral counseling may be effective in reducing daily smoking and other associated smoking behavior. Although both training types (and to some extent both sham conditions) seemed to exert beneficial effects on behavior, our results provide tentative hints for the fact that avoidance training might be somewhat more effective in reducing daily smoking than inhibition training. However, this effect was not long-lasting and awaits further replication. Some training effects could be established (i.e., strengthened approach biases for control pictures presented during training, reduced Stroop interference effect), which, however, were independent of training condition. Further research is required to validate the current findings and to identify the precise mechanisms underlying the effects of the training variants on tobacco smoking.

## Supplementary Information

Below is the link to the electronic supplementary material.

Supplementary file1 (PDF 829 KB)
