# Supplementary material for: “Push it!” or “Hold it!”? A comparison of nicotine-avoidance training and nicotine-inhibition training in smokers motivated to quit
Source: Psychopharmacology (Berl). 2022 Jan 11;239(1):105–21. doi: 10.1007/s00213-021-06058-5 (PMC8748000; doi:10.1007/s00213-021-06058-5)
Supplement: Supplementary file 1 — Supplementary file1 (PDF 829 KB) [file 213_2021_6058_MOESM1_ESM.pdf]

Supplementary Information (IS)

Here, we report detailed descriptions of experimental tasks used for cognitive bias assessment and self-report data. We also report findings related to a number of secondary hypotheses that were not covered in the primary manuscript, along with additional Tables and Figures.

Methods

Motor approach biases for smoking (training effects and close generalization). Prior to the first (pretest) and upon the last (posttest) training session, all smokers completed an assessment version of the AAT (based on Machulska et al. 2016). The task started with 12 practice trials comprising neutral stimuli, which were not included in the final analyses. For the purpose of bias assessment, 15 nicotine-related and 15 tooth-cleaning (control) pictures had to be pulled and pushed equally often and were presented in a quasi-random order (at most three equal rotations or picture categories in a row). Two-thirds of the pictures of the assessment AAT were also used in the training-AATs (10 smoking-related and 10 tooth-cleaning pictures). The remaining pictures were not presented during training, which was done to allow for a test of close generalization effects. Images were rotated either 3° to the left or 3° to the right. Similar to the training, participants were instructed to pull images rotated to the left and to push images rotated to the right. Each image was presented three times in pull-closer and three times in push-away format, resulting in 180 test trials. In addition, participants randomized to the A-AAT (active or sham condition) completed a brief approach bias assessment consisting of 40 test trials prior to each training session. An approach bias was inferred from faster pulling than pushing a picture. An approach bias score was calculated for each participant and picture category by subtracting the median reaction time (RT) for pulling the picture category from the median RT for pushing the picture category (see Rinck and Becker 2007). By doing so, a positive score indicates an approach tendency toward a picture category, whereas a negative score indicates an avoidance tendency.

Approach associations for smoking (broad generalization). All participants completed a single-target Implicit-Association-Test (st-IAT) at pre- and posttest. The st-IAT was based on Woud and colleagues (2016). The task started with an attribute discrimination block, in which participants had to categorize approach- or avoidance-related words by pressing a keyboard key (i.e., press “A” for approach words, press “K” for avoidance words). In total, six different approach and avoidance words were presented in a fixed random order. The first combined block (24 practice + 72 test block trials) added six different smoking words. During the compatible block assignment, smoking and approach-related words shared a response key, while in the incompatible block (24 practice + 72 test block trials), smoking and avoidance-related words shared the same

response key. During each trial, reminder labels remained visible on the computer screen. Upon incorrect responses, a red “X” appeared in the center of the screen. Response assignments and block order were counterbalanced across participants. Approach associations were inferred from faster reactions to trials in which smoking and approach words shared a response key as opposed to trials where smoking and avoidance words shared a response key. As such, an approach bias was calculated by subtracting the median RT of the compatible block from the median RT of the incompatible block. Accordingly, a positive score reflects stronger approach associations toward smoking words.

Nicotine-related response inhibition (close generalization). Each GNG training session started with 40 test trials, in which smoking and control images were equally often presented in go- and no-go format. Hence, participants had to respond or withhold their response to nicotine-related and control pictures with equal frequency. The tendency to more rapidly respond to smoking pictures was investigated by analyzing median go-RTs. According to Di Lemma and Field (2017), a GNG-Bias can be inferred from subtracting the RTs for go-smoking trials from those of go-control trials. A positive score indicates that smokers are faster to respond to smoking pictures than to control pictures, suggesting an automatic tendency to react to smoking cues or reduced response.

Inhibition of habitual responses (broad generalization). All participants performed the classical Stroop-Task (Stroop 1935), which was adapted from Hepp and colleagues (1996). Stimuli consisted of color words (German equivalents for “red”, “green”, “blue”, and “black”) and neutral objects (rectangles), all of which were printed in either red, green, blue, or black. Participants were required to ignore the semantic meaning of the words and instead indicate the print color by responding via keyboard button presses (“d” for red, “f” for green, “j” for blue, “k” for black). During each trial, reminder labels remained visible on the computer screen. Upon incorrect responses, a red “X” appeared in the center of the screen. During congruent trials (28 trials), color words appeared in their corresponding color (e.g., the word “red” printed in red ink). In incongruent trials (28 trials), color words were printed in a non-matching color (i.e., the word “red” printed in black). Neutral control trials (28 trials) consisted of displaying colored rectangles, lacking potential matches or mismatches between semantic and visual proceeding. Stimuli were presented in a random order. Following Hepp and colleagues (1996), two different Stroop-Scores were calculated: An interference effect was calculated as the difference between the median RTs of incongruent trials and neutral control trials.

Self-report data and bio-chemical verification

Nicotine consumption and expired carbon monoxide (CO). Participants indicated the number of cigarettes smoked daily at pre-, posttest, FU-4, and FU-12. Abstinence was assessed at posttest and both FUs via self-report. In addition, exposure to nicotine was measured by means of a CO breath test (piCO™ Smokerlyzer®; Bedfont Scientific Ltd) at pre- and posttest.

Craving. Craving was assessed on a 6-point Likert scale, ranging from 0 (“not at all”) to 5 (“very high). Assessment times included pretest, prior to each training session (t1, t2, t3, t4, t5) and posttest.

Nicotine dependence. The degree of nicotine dependence was measured by means of the Fagerström Test for Nicotine Dependence (FTND; Heatherton et al. 1991; German version: Bleich et al. 2002). The FTND consists of six items with a sum score ranging between 0 (no/very weak dependence) and 10 (very strong dependence). The FTND was administered at pretest, posttest, FU1, and FU2.

Motivation to cease smoking. Based on the transtheoretical model of change (Prochaska and DiClimente 1982), a Stages of Change Scale (SoC) was applied (Prochaska et al. 1991; German version: Jäkle et al. 1994). The two-item questionnaire assigns smokers to different time intervals of change: precontemplation, contemplation, preparation, action, or maintenance. In addition, the Thoughts About Abstinence Scale (TAA; Hall et al. 1990) asked participants to select one of six abstinence goals: (a) total abstinence, never use again, (b) total abstinence, but realize a slip is possible, (c) occasional use when urges strongly felt, (d) temporary abstinence, (e) controlled use, and (f) no goal. Subsequently, patients rated (a) their desire to quit, (b) the expected success in quitting, and (c) the expected difficulty of quitting on 10-point Likert-scales. Both measures (SoC; TAA) were administered at pre- and posttest.

Psychopathology. To control for preexisting differences in substance use behavior other than smoking and in mental health, and to monitor possible adverse training effects, the Alcohol Use Disorders Identification Test (AUDIT; Saunders et al. 1993) and the Depression-Anxiety-Stress-Scale 21 (DASS 21G; Lovibond and Lovibond, 1995) were administered at pre- and posttest.

Devaluation

Picture evaluation (close generalization). At pre- and posttest, participants evaluated a selected set of stimuli presented during approach bias assessment (six nicotine-related and six tooth-cleaning picture) according to three different criteria: valence (positive vs. negative), arousal (calming vs. arousing), and craving (highly triggering craving vs. not at all triggering craving). Here, 10-point Likert scales were applied. Two-thirds of the

pictures were also presented during training, while the remaining pictures were not. This approach allows for a test of close generalization effects of AAT-training on subjective stimulus evaluations.

Smoking attitudes (broad generalization). Explicit attitudes toward smoking were measured via a set of eight semantic differential items, which were based on Swanson and colleagues (2001). A 7-point Likert scale was used to rate 8 different polar-opposite adjective pairs (i.e., healthy-unhealthy). Each scale ranged from -3 to +3 with greater values being associated with more positive attitudes toward smoking.

Results

Cigarette Craving

In Figure S2, we see similar rebound effects for the A-AAT, sham A-AAT and sham GNG-AAT groups. For the GNG-AAT training, the rebound effect appears to be less pronounced. This effect, however, was not statistically significant.

Abstinence

Data for abstinence were available from the post-assessment and the two follow-up examinations. Probability of nicotine abstinence was analyzed by a binomial generalized linear mixed effects model (random intercept model). Table S4 shows model results on the logit scale. Predicted probabilities (in percent) for being abstinent are displayed in Figure S4. The relatively large standard errors suggest quite a large within-group heterogeneity. Neither effects for time (for follow-up 1: $t(224.32)=.72$; $p = .473$; for follow-up 2: $t(178.75)=.87$; $p = 387$), nor experimental condition ($t(212.52)=-1.93$; $p = .054$) could be found. A descriptive analysis of the predicted probabilities suggests that the average abstinence appears to increase over time, especially in the experimental conditions A-AAT and GNG-AAT, as well as the sham GNG-AAT. While at the post measurement predicted probabilities for abstinence only ranged between 4.07% and 11.72%, probabilities ranged between 7.89% and 32.19% at the second follow-up examination. Lowest abstinence rates were predicted for the sham A-AAT.

Appendix

Table S1. Reliability estimates of cognitive bias measures

Table S2. Changes in daily cigarette smoking

Table S3. Changes in cigarette craving

Table S4. Abstinence

Table S5. Estimated Marginal Means of other smoking-related variables

FigA1 Changes in expired CO

FigA2 Changes in daily cigarette craving

FigA3 Changes in nicotine dependence

FigA4 Predicted probabilities for abstinence

FigA5 Changes in GNG-Biases

FigA6 Predicted marginal means for the Stroop interference effect

FigA7 Stimulus evaluation regarding valence, arousal, and craving

Table S1. Reliability estimates of cognitive bias measures

Paradigm	Items/ category	Timepoint						
**AAT**		Pretest	T1	T2	T3	T4	T5	Posttest
Smoking approach bias (trained)	10	.527	.164	.391	.251	.491	.041	.548
Smoking approach bias (untrained)	5	.010	-	-	-	-	-	.498
Control approach bias (trained)	10	.497	.253	.052	.314	.032	.310*	.574
Control approach bias (untrained)	5	.049	-	-	-	-	-	.043
**st-IAT**								
Smoking approach associations	6	.766	-	-	-	-	-	.774
**Go/No-Go-Task**								
Go/No-Go-Bias	10	-	-.024	-.157	-.096	-.030	.143	-
**Stroop-Task**								
Stroop-Inference Score	4	.710	-	-	-	-	-	.546
Stroop-Facilitation Score	4	-.161	-	-	-	-	-	.418

Note. Reliability was estimated by means of internal consistency (Cronbach's α). T1 = Training 1, T2 = Training 2, T3 = Training 3, T4 = Training 4, T5 = Training 5; AAT = Approach-Avoidance-Task; st-IAT = single-target Implicit Association Test; *one item was excluded from the analysis due to negative correlations with all other items.

Table S2. Changes in daily cigarette smoking

	EST	SE	t	df	p	FMI
I	2.04	0.23	8.81	655.40	0.000	0.02
S	-0.54	0.17	-3.15	592.99	0.002	0.08
Q	0.08	0.03	2.30	553.32	0.022	0.11
I on GNG-AAT	-0.31	0.32	-0.97	654.98	0.333	0.02
I on sham A-AAT	0.40	0.33	1.23	653.52	0.220	0.02
I on sham GNG-AAT	0.28	0.33	0.84	659.76	0.399	0.01
S on GNG-AAT	0.17	0.24	0.72	570.55	0.470	0.10
S on sham A-aat	0.15	0.25	0.60	536.22	0.551	0.13
S on sham GNG-AAT	-0.08	0.25	-0.34	584.29	0.737	0.09
Q on GNG-AAT	-0.05	0.05	-1.08	545.75	0.280	0.12
Q on sham A-AAT	-0.04	0.05	-0.73	497.45	0.468	0.16
Q on sham GNG-AAT	0.01	0.05	0.11	553.82	0.913	0.11

Note. EST is the combined estimate based on m=100 imputations, SE the corresponding standard error, FMI the estimated fraction of missing information. Coefficients are described from a structural equation model (SEM) perspective. I = intercept, S = slope, Q = quadratic time trend; I, S and Q denote the baseline level of cigarettes smoked per day (intercept), the linear (slope) and quadratic time trend respectively. “I on condition” gives an indication of baseline group differences. “S on condition” and “Q on condition” are interaction between different patterns of time (linear or quadratic) and condition. These quantities are regressed on experimental condition.

Table S3. Changes in cigarette craving

	EST	SE	t	df	p	FMI
I	2.04	0.23	8.81	655.40	0.000	0.02
S	-0.54	0.17	-3.15	592.99	0.002	0.08
Q	0.08	0.03	2.30	553.32	0.022	0.11
I on GNG-AAT	-0.31	0.32	-0.97	654.98	0.333	0.02
I on sham A-AAT	0.40	0.33	1.23	653.52	0.220	0.02
I on sham GNG-AAT	0.28	0.33	0.84	659.76	0.399	0.01
S on GNG-AAT	0.17	0.24	0.72	570.55	0.470	0.10
S on sham A-AAT	0.15	0.25	0.60	536.22	0.551	0.13
S on sham GNG-AAT	-0.08	0.25	-0.34	584.29	0.737	0.09
Q on GNG-AAT	-0.05	0.05	-1.08	545.75	0.280	0.12
Q on sham-AAT	-0.04	0.05	-0.73	497.45	0.468	0.16
Q on sham GNG-AAT	0.01	0.05	0.11	553.82	0.913	0.11

Note. See Table S2.

Table S4. Abstinence

	EST	SE	t	df	p	FMI
(Intercept)	-2.02	1.04	-1.93	212.52	0.054	0.25
t2	0.69	0.96	0.72	224.32	0.473	0.23
t3	0.89	1.03	0.87	178.75	0.387	0.33
GNG-AAT	-0.79	1.28	-0.62	245.50	0.536	0.18
Sham A-AAT	-1.14	1.27	-0.90	298.56	0.369	0.07
Sham GNG-AAT	-0.48	1.25	-0.39	280.08	0.700	0.11
t2 x GNG-AAT	0.46	1.36	0.34	202.66	0.735	0.27
t3 x GNG-AAT	0.67	1.35	0.49	209.19	0.623	0.26
t2 x sham A-AAT	-0.12	1.39	-0.09	245.24	0.929	0.18
t3 x sham A-AAT	-0.19	1.51	-0.13	185.67	0.898	0.31
t2 x sham GNG-AAT	0.13	1.35	0.09	220.28	0.926	0.23
t3 x sham GNG-AAT	0.86	1.39	0.62	199.74	0.537	0.28

Note. See Table S2.

Table S5. Estimated Marginal Means of other smoking-related variables

	T1				T2			
	**A-AAT**	**GNG-AAT**	**Sham A-AAT**	**Sham GNG-AAT**	**A-AAT**	**GNG-AAT**	**Sham A-AAT**	**Sham GNG-AAT**
days without cigarettes	3.67	7.41	3.58	4.12	6.51	13.71	7.07	8.14
attitude	4.40	5.00	4.49	4.78	5.18	5.50	4.98	5.41
SoC1	1.36	1.48	1.36	1.41	1.81	1.68	1.83	1.61
SoC2	1.11	1.58	1.29	1.89	1.32	1.29	1.53	1.72
abstinence (goal)	4.07	4.10	3.89	3.85	4.28	4.21	3.92	3.51
abstinence (desire)	8.75	8.32	8.61	8.33	8.83	8.29	8.01	8.40
success	6.14	5.97	6.04	6.48	6.69	7.02	6.30	6.40
difficulties	8.21	6.97	8.11	8.30	6.60	6.47	6.53	7.82
AUDIT	5.50	4.29	7.36	7.07	4.87	3.89	6.18	5.89
DASS stress	6.86	5.68	6.50	5.26	5.38	4.71	5.34	4.72
DASS anxiety	3.46	3.06	4.18	3.22	2.58	2.10	2.50	2.10
DASS depression	4.39	3.06	4.71	4.26	3.30	2.78	2.70	2.62

Note. For ‘days without cigarettes’ T1 and T2 denote the first and second follow-up measurement, for all other variables pre and post measurement, respectively.

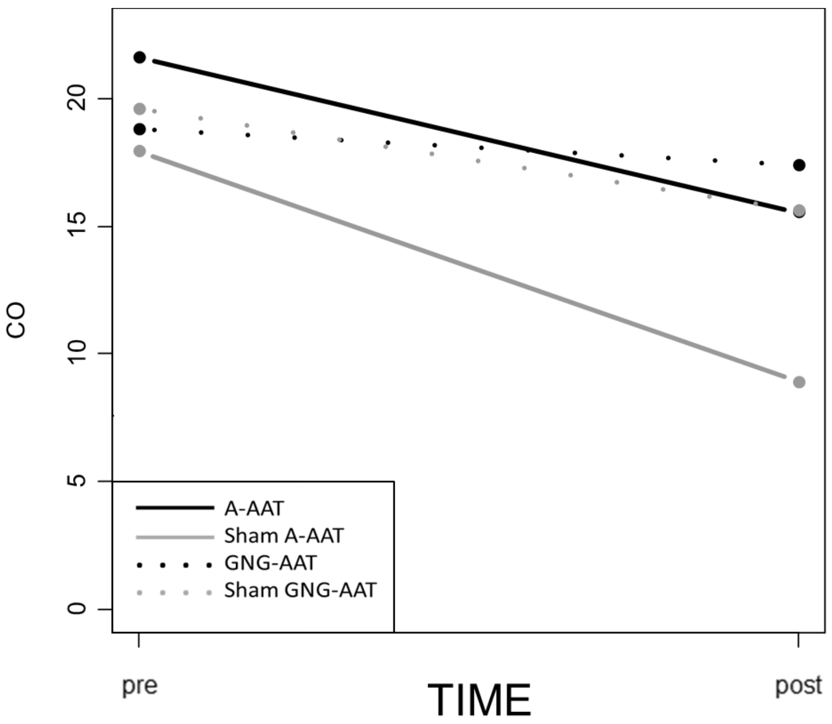

Fig A1 Changes in expired CO

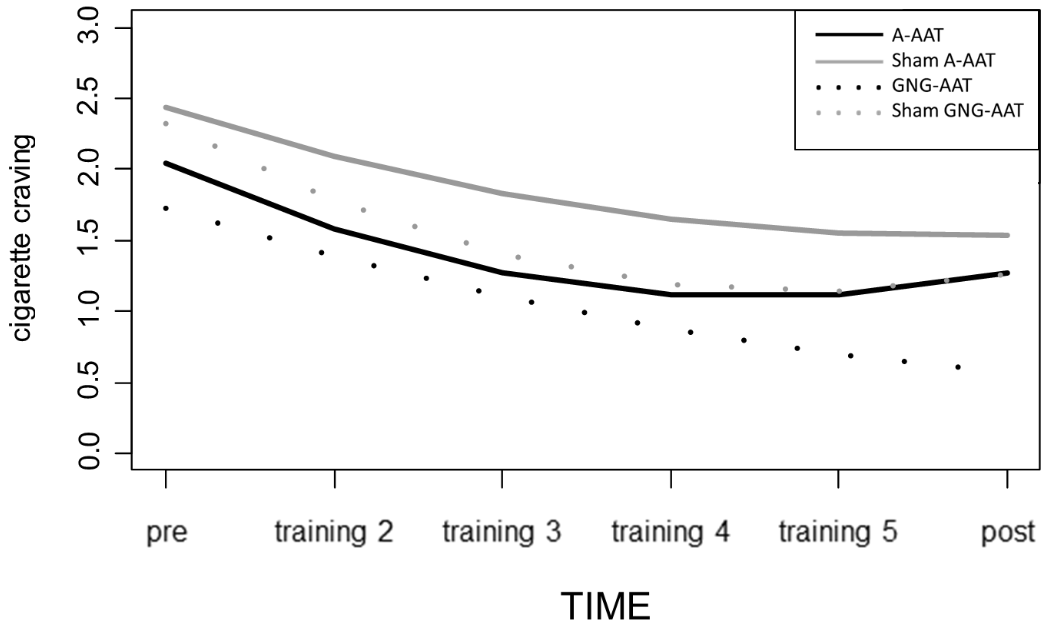

FigA2 Changes in daily cigarette craving

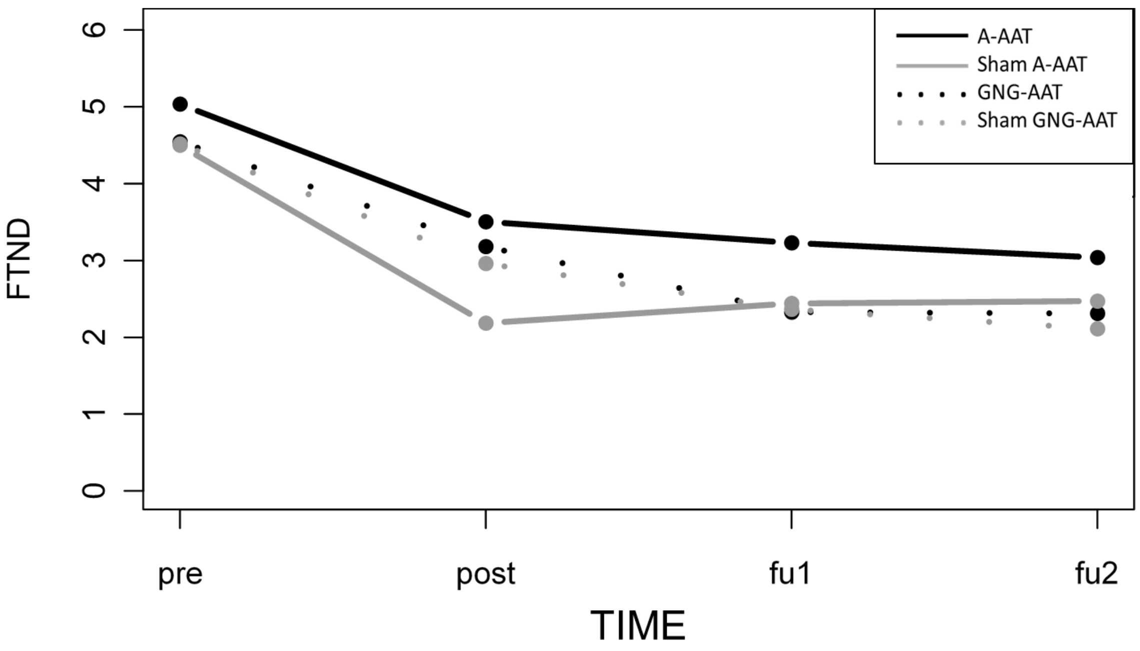

FigA3 Changes in nicotine dependence

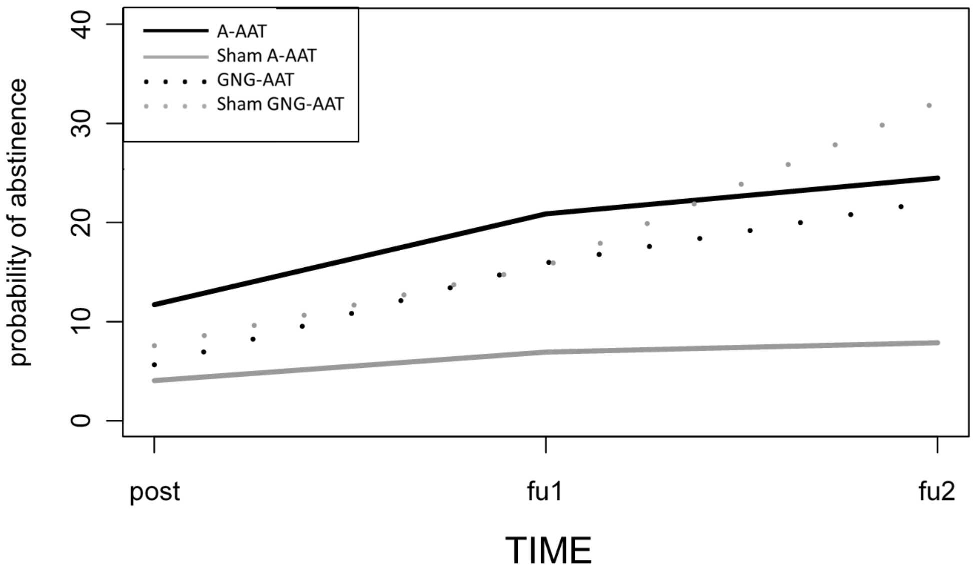

FigA4 Predicted probabilities for abstinence

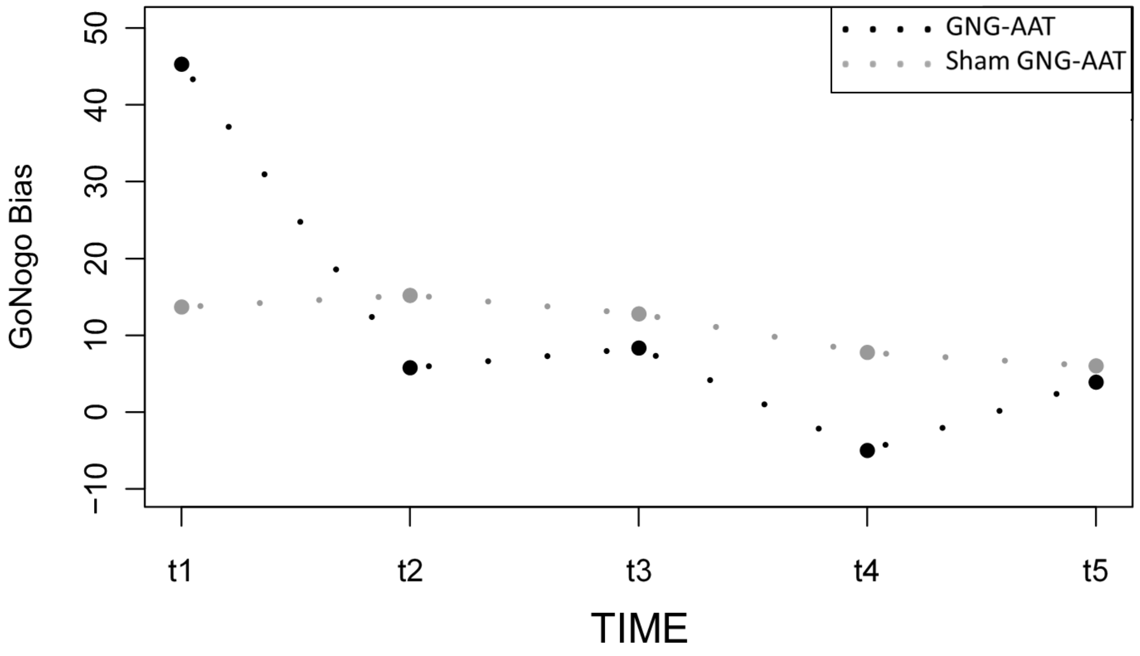

FigA5 Changes in GNG-Biases

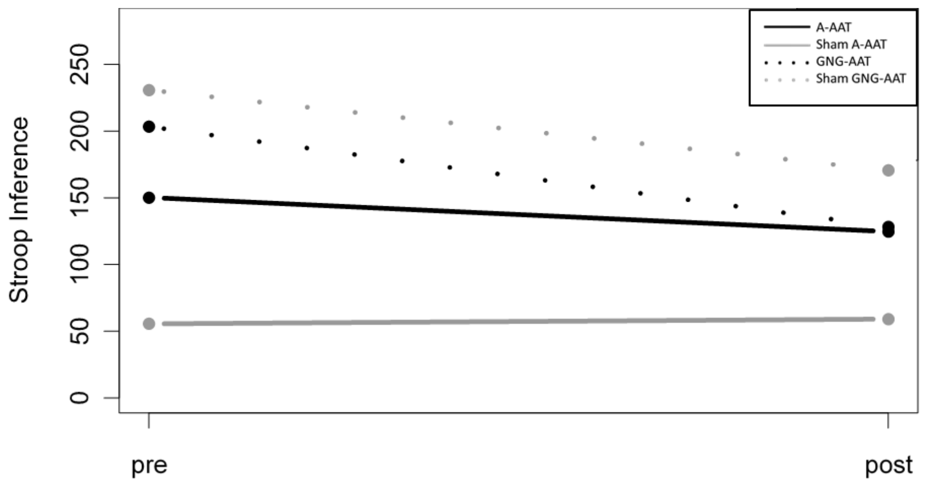

FigA6 Predicted marginal means for the Stroop interference effect

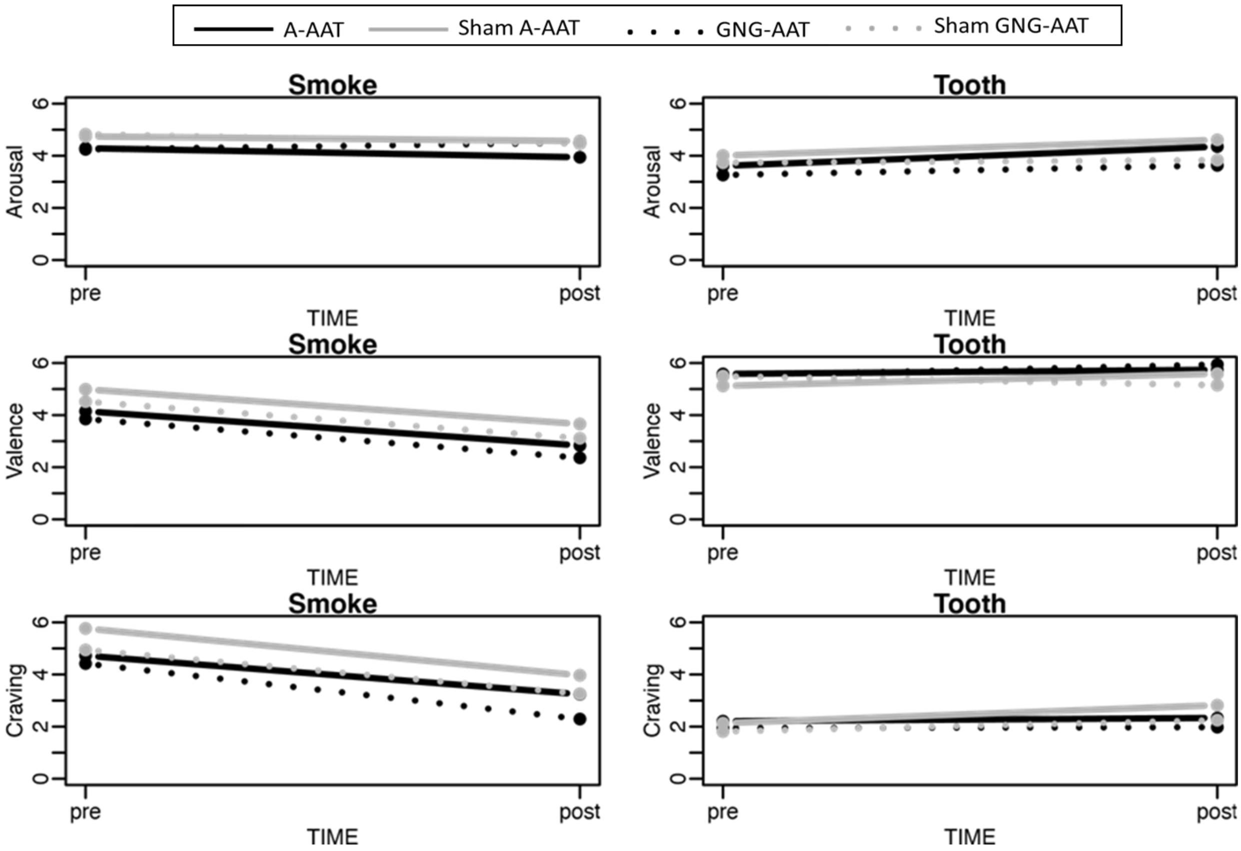

Fig A7 Stimulus evaluation regarding valence, arousal, and craving
